# The Feasibility of RGB-D Gaze Intention Measurement in Children with Autism Using Azure Kinect

**DOI:** 10.3390/bioengineering12040370

**Published:** 2025-04-01

**Authors:** Abderrahmen Bendimered, Rim Cherif, Rabah Iguernaissi, Mohamad Motasem Nawaf, Susanne Thümmler, Séverine Dubuisson, Djamal Merad

**Affiliations:** 1 Laboratoire d’Informatique et des Systèmes, CNRS UMR 7020, Université de Marseille, Aix-Marseille University, 13009 Marseille, France; rim.cherif@lis-lab.fr (R.C.); rabah.iguernaissi@lis-lab.fr (R.I.); motasem.nawaf@lis-lab.fr (M.M.N.); severine.dubuisson@lis-lab.fr (S.D.); djamal.merad@lis-lab.fr (D.M.); 2 University Department of Child and Adolescent Psychiatry, Children’s Hospitals of Nice CHU Lenval, 06103 Nice, France; susanne.thummler@univ-cotedazur.fr; 3 CoBTek, Université Côte d’Azur, 06103 Nice, France

**Keywords:** autism spectrum disorder, gaze tracking, RGB-D technology, computer vision

## Abstract

Gaze interpretation is a fundamental aspect of social communication, especially for children with autism spectrum disorder (ASD), who frequently encounter many difficulties in social interactions. Despite the considerable advances made in gaze tracking technologies, such as those based on RGB and RGB-D, the accurate measurement of gaze direction remains a significant scientific challenge. This paper proposes a novel approach utilizing the Azure Kinect to improve the measurement of gaze intention in children with ASD, providing accurate estimations of both gaze direction and head position. To evaluate the effectiveness of the proposed methodology, an experimental trial was conducted with eight participants of varying statures. The intersection of the estimated gaze with the target plane was also analyzed throughout 38-min sessions. The results demonstrated high accuracy, with a minimum angular error of 2.5° using pupil positions, 2.06° using head orientation, and average errors of 4.46° and 3.19°, respectively. This approach was tested on a dataset of children with ASD to track their gaze towards a clinician, as this information is essential for assessing the children’s social intent and interactions with others, facilitating more precise clinical assessments for children with autism.

## 1. Introduction

Eye tracking is an important tool for understanding cognitive processes, attention, and behavior. It has been adopted in several fields such as VR [1], HCI [2], and monitoring systems such as fatigue detection [3]. Eye movement tracking provides valuable insights into how people interact with their environment. For example, recent studies [4] have demonstrated how eye tracking, combined with machine learning, can classify complex cognitive dimensions such as self-orientation, goal orientation, and sensory modalities.

Eye tracking plays a crucial role in detecting and understanding ASD. Atypical gaze behaviors, such as reduced eye contact, are often among the earliest signs of ASD, and can sometimes be observed as early as six months of age. Early detection is crucial, as it can significantly improve the care provided to children with ASD. Computer vision-based eye tracking can become a key tool to identify these atypical gaze patterns, potentially reducing reliance on traditional methods and aiding in difficult diagnostic procedures such as ADOS [5]. In addition, tracking eye movements can provide valuable insights into the neurological and social aspects of ASD, enhancing our understanding of how people with ASD perceive and interact with the world. Furthermore, recent work by [6] proposed a gamified web system for data collection, combining crowdsourcing algorithms with machine learning to assist in diagnosing developmental delays such as ASD, aiming to improve the precision of early diagnosis.

Given the complex and challenging nature of ASD diagnosis, automating some aspects of the diagnostic process could significantly improve its efficiency and accuracy. Eye-tracking technology can assist clinicians by providing objective, real-time data on gaze patterns. These data can be used to detect ASD signs, reducing reliance on subjective human interpretations and making the diagnostic process more consistent and scalable. For example, algorithms can be developed to automatically analyze eye movement patterns and identify potential cases of ASD, helping clinicians in the diagnostic decision-making process.

Several technologies are used to capture eye movements. Traditional systems based on electro-oculography (EOG), which measures electrical potentials around the eyes, are robust to changes in lighting and can provide real-time data. However, their sensitivity to head movements and intrusive nature limit their applicability [7,8,9]. Another common method is scleral search coils, which use magnetic fields to track small coils attached to the surface of the eye. While these systems provide highly accurate results, their invasive nature restricts their use to controlled environments [10,11].

In contrast, non-invasive camera-based systems have become increasingly popular due to their ability to capture natural eye movements. These methods fall into two main categories: model-based and appearance-based approaches. Model-based methods use geometric models of the eye and head, often augmented by lighting systems such as infrared LEDs or RGB-D cameras to estimate 3D gaze direction [12,13]. Appearance-based methods, on the other hand, use deep learning models to map facial and eye features to gaze direction. These methods benefit from large annotated datasets such as EYEDIAP, UnityEyes, and MPIIGaze [14,15,16]. Hybrid approaches, combining both model-based and appearance-based techniques, have been shown to be more robust in dynamic environments [17].

In modern gaze tracking, two main approaches are commonly used: RGB-based and RGB-D-based methods [18]. RGB-based methods, which rely on standard image datasets and deep learning models, have made significant advances in accuracy, but have difficulty estimating depth, and maintain precision under dynamic conditions such as changing head poses and lighting [19]. RGB-D methods, which integrate depth data, offer a significant advantage by improving robustness to these environmental challenges. For example, recent work has shown that combining RGB-D sensors with neural networks allows accurate 3D gaze estimation, even under low light conditions [20].

Despite these advances, eye-tracking faces challenges in real-world conditions due to variations in lighting, head motion, and facial morphology, which introduce errors and reduce robustness. These challenges are particularly critical in ASD diagnosis, where systems need to adapt to different user characteristics, such as the use of glasses.

In this paper, we propose a novel real-time RGB-D-based gaze tracking method with the Azure Kinect camera [21], which uses 3D anatomical landmarks such as head orientation and iris positions to accurately compute gaze direction and facilitate the automatic measurement of intentions in children with autism. The key contributions of our work are as follows: (1) An innovative gaze estimation that combines 3D iris and head position data to enable accurate real-time gaze tracking; (2) Robust performance, reliable at distances up to 3.4 m, even when eye details are less visible; (3) An application in ASD research by analyzing interactions between children with ASD and clinicians to gain insight into atypical behaviors and therapeutic responses. To validate our method, we conducted experiments with eight participants under different conditions, measuring the angular error between the gaze centroid and the target on a 2D plane. The results demonstrated the robustness and accuracy of our approach, highlighting its potential for applications in ASD diagnosis and therapy.

The remainder of this paper is organized as follows: Section 2 describes the data collection process, including the 3D gaze data acquisition protocols and details of the ASD dataset, as well as the methodology for 3D iris- and head-based gaze estimation. Section 3 presents the experimental results, including performance evaluation, accuracy metrics, and the application of our approach to ASD research, providing a summary of the results and discussion. Section 4 presents the conclusion, which summarizes the findings of the study and discusses the implications for gaze-tracking research and ASD interventions.

## 2. Experimental Setup and Methodology

### 2.1. Data Collection

Data were collected as part of an experimental protocol involving eight adult participants. During each session, participants focused on fixed targets on a board, with variation introduced by changing their distance from the targets. Each session lasted approximately 38 min, including breaks and tests, for a total recording time of 46.6 min (8 participants × 5 positions × 7 targets × 10 s per target). A total of 83,880 frames were recorded at 30 FPS. The participants were adults aged 25 to 33, consisting of five males and three females.

The eight participants in this phase were neurotypical adults. This preliminary stage aimed to evaluate the technical reliability of our gaze estimation method in a 3D reference world before applying it to autistic children. Since autistic children may experience challenges performing certain tasks, we conducted this initial validation with adults.

We validate our approach using patient recordings, employing two estimation methods: (1) Eye-based estimation—this determines the center of the eye using detected eye landmarks. And (2) Head orientation-based estimation—this utilizes a 3D skeletal model and detects head joints. Since our method is based on biomechanical gaze detection, specifically through eye landmarks, it remains independent of age or cognitive development. Therefore, whether the participant is an adult or a child does not affect its validity.

Seven targets were marked on a board placed parallel to the X−Y plane of the camera at a distance of 398.3 cm from the camera. The Azure Kinect camera was positioned at a height of 144.2 cm. The 3D coordinates of these targets in the RGB sensor coordinate system are shown in Table 1, which outlines the *X*, *Y*, and *Z* coordinates for each target. This configuration was maintained for both studies.

Participants were placed in front of the camera at five different locations. The corresponding coordinates for each position are shown in Table 2, which lists the *X*, *Y*, and *Z* coordinates for each participant’s position.

**Figure 1:**
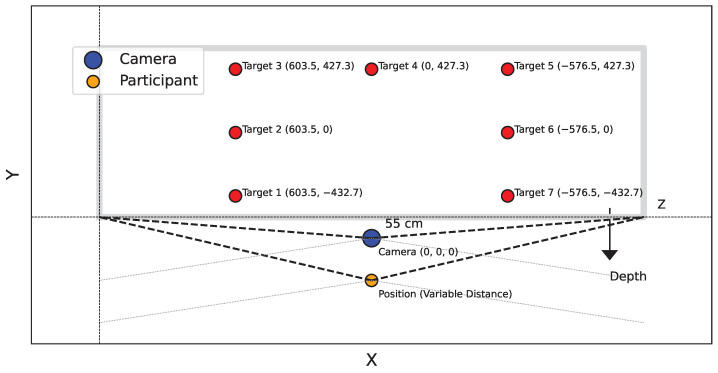
Experimental setup and workflow for 3D landmark detection and iris tracking.

The experimental setup is summarized in Figure 1. The Azure Kinect sensor provides a variety of data, including joint coordinates for human skeletons with confidence score for each detected joint. For this study, we focused on the 3D coordinates of the ears, nose, and eyes. The 2D-3D conversion was performed using calibration data from the Azure Kinect sensor. To detect the iris data that are not provided by the Kinect, we chose the GazeML [22] method. To evaluate the performance of this landmark detection model, we tested it with three other different methods on a 30-min recording (consisting of three 10-min sequences, each with a single subject). This recording contained approximately 54K frames at 30 FPS. The goal of the evaluation was to measure the number of images with successful detection in order to determine the most reliable model for our study. We chose this method because of its ability to accurately estimate the position of the eyes and iris even under difficult conditions, such as extreme head orientations or the presence of glasses.

Lighting conditions were systematically varied throughout the sessions to account for natural daily fluctuations, including weather-related changes. Additionally, differences in participant height affected the relative position of the Azure Kinect camera. These variations were introduced to assess the robustness of our method and its ability to function reliably in real-world environments, where such factors naturally fluctuate.

In the adult acquisition tests, we initially used distance thresholds ranging from 55 cm to 340 cm. These distances were selected to validate our method under conditions resembling clinical consulting rooms, where data collection for autistic children took place. Since the room dimensions in both settings were comparable, this range ensures that our method remains robust and effective for application in real clinical environments.

This database is used to validate both gaze detection and head orientation components. The eight adult participants completed the designated protocols, which were complex and physically demanding. It is important to note that individuals with autism spectrum disorder (ASD) may face challenges completing all tasks due to their behavioral and cognitive characteristics. Therefore, we conducted this technical validation phase with neurotypical adults before applying the algorithm to data from autistic children.

The autistic children’s dataset was collected over two sessions, each of which involved a child interacting with a clinician in a dedicated room. Each session lasted approximately an hour and was recorded using four Azure Kinect sensors. These sensors captured RGB images, depth images, and 3D coordinates of the human skeleton for each person in the room. The RGB images were recorded at a resolution of 1280 × 720 pixels, while the depth images were recorded at a resolution of 320 × 288 pixels. The recordings were conducted at 30 frames per second to ensure high temporal resolution for analysis. These recordings are part of the ACTIVIS database, as detailed in [23]. Table 3 provides annotations on gaze durations, with Session 1 corresponding to Child 1 and Session 2 corresponding to Child 2. Each period in the table represents the times when the child looked at the clinician and was actively engaged in an interaction with them. For each period, the annotation indicates the exact moment when the child began to look at the clinician and the moment when the child’s gaze turned away.

**Figure 2:**
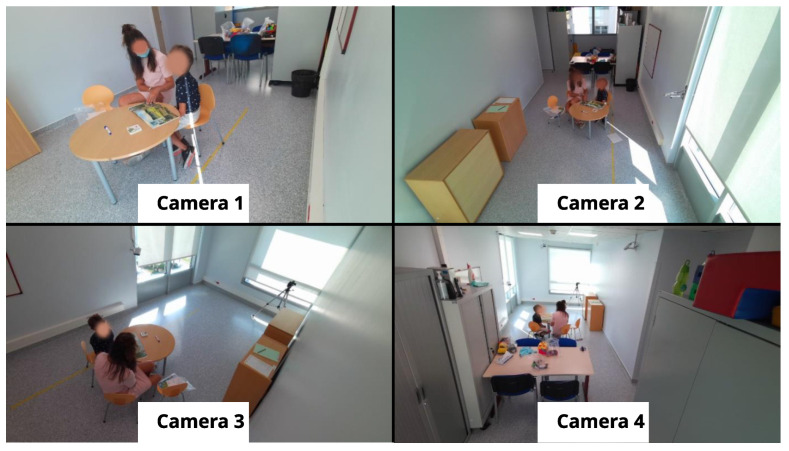
Annotated frame of Child 1 looking at the clinician (multi-camera views).

**Figure 3:**
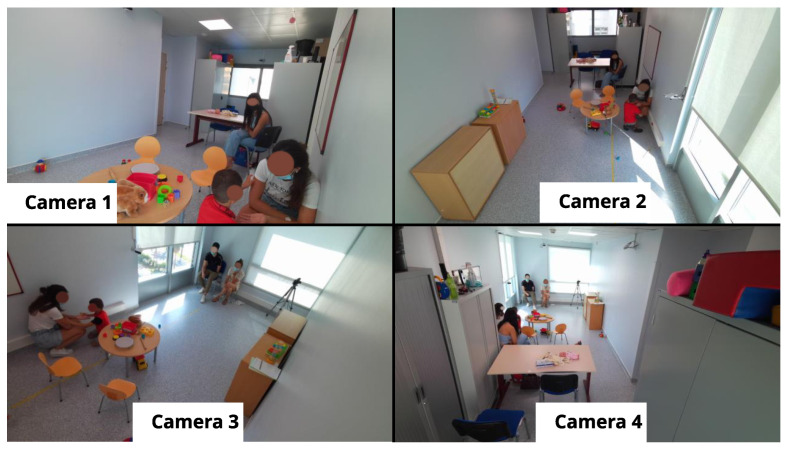
Annotated frame of Child 2 looking at the clinician (multi-camera views).

Figure 2 shows an example of an annotated frame where Child 1 is looking at the clinician, while Figure 3 shows a similar annotation for Child 2. These figures also show views captured simultaneously by all four cameras, providing a comprehensive perspective of the interactions from multiple angles.

#### Ethical Considerations

This study was approved by the CERNI Ethics Committee under approval code 2020-04-005, granted on 5 April 2020. Written informed consent was obtained from all participants, as well as from the parents or legal guardians of the children involved. Data collection and processing adhered to national and international ethical guidelines, ensuring anonymity and confidentiality of all information.

### 2.2. Evaluating Gaze Direction in Children with ASD

**Figure 4:**
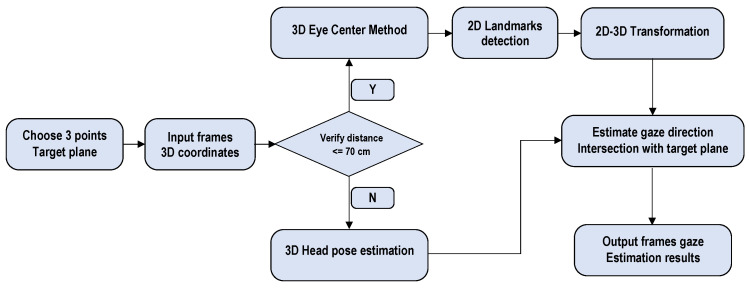
Workflow for real-time gaze direction estimation.

This section describes the process of gaze direction estimation in children with ASD. The workflow shown in Figure 4 outlines the steps involved in real-time gaze estimation.

The workflow is designed to capture and analyze gaze behavior with a focus on children with ASD:

- Initialization: Three points are defined to establish the target plane.
- Input Data Acquisition: 3D coordinates are captured from input frames using devices such as Azure Kinect to provide 3D data.
- Distance Verification: A threshold of 70 cm is used to verify that the subject is positioned at a distance less than or greater than 70 cm from the camera. Based on this condition, the application selects the appropriate gaze estimation method.
- If the distance <= 70 cm:
  − Three-dimensional Eye Center Method: This method determines gaze direction using anatomical and optical axes for increased accuracy.
  − Landmark Detection and 3D Transformation: 2D eye landmarks are detected and transformed into 3D coordinates using the Azure Kinect feature.
- When the distance > 70 cm:
  − Head Pose Estimation: The head pose is calculated from the 3D coordinates of the face landmarks.
- Eye direction estimation: The intersection of the gaze direction with the target planes is computed to determine the subject’s gaze position.
- Output frames: Real-time gaze estimation results are provided.

This method was chosen for its ability to improve gaze tracking accuracy, particularly in children with ASD, whose eye movements can be more difficult to analyze.

### 2.3. 3D Gaze Estimation Based on Eye Center

**Figure 5:**
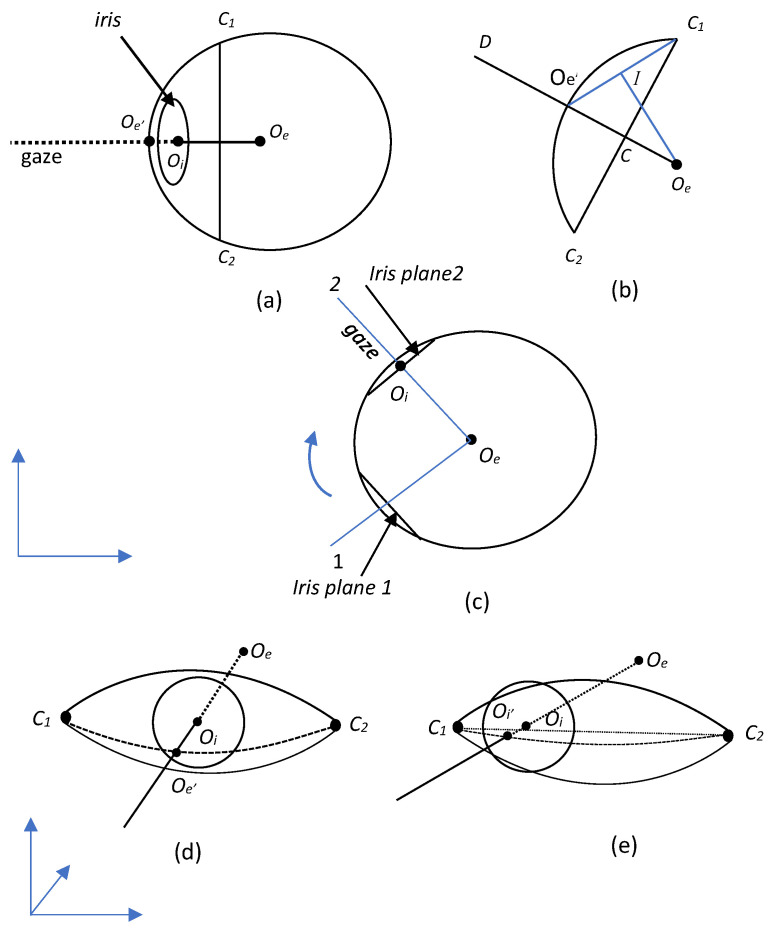
Steps of 3D gaze estimation, (**a**) The eye model is made up of the center of the eyeball $O_{e}$ and the center of the iris $O_{i}$ with projections and geometric relations between them. (**b**) The rotation of the eye is modeled by the anatomical axis passing through the center of the eye and the iris. (**c**) The direction of gaze is shown by a line from the eye center to the iris. The 2D coordinates are transformed to 3D for real-time gaze tracking. (**d**) The eye center coordinates are used to determine the gaze direction. (**e**) The iris center is projected onto the eye surface.

Figure 5a shows a simplified model of the eye, where the eyeball is represented as a sphere with its center at $O_{e}$. The iris is represented as a circle with its center at $O_{i}$, and the point $O_{e}^{\prime }$ is the orthogonal projection of $O_{e}$ onto the surface of the sphere Figure 5b. The rotation of the eye is modeled by the anatomical axis passing through the center of the eye and the iris; see Figure 5c.

For a circle defined by three points, including $C_{1}$ and $C_{2}$, we have the following relations:

(1) $$O_{e^{\prime }}C_{1}^{2}=O_{e^{\prime }}C^{2}+\frac{C_{2}C_{1}^{2}}{4}\;\mathrm{Pythagorean}\;\mathrm{Theorem}$$

(2) $$\frac{O_{e^{\prime }}I}{O_{e^{\prime }}C}=\frac{O_{e^{\prime }}O_{e}}{O_{e^{\prime }}C_{1}}\;\mathrm{similar}\;\mathrm{triangles}$$

(3) $$O_{e^{\prime }}O_{e}^{2}=\frac{O_{e^{\prime }}C_{1}^{2}}{2\times O_{e^{\prime }}C}$$

(4) $$O_{e^{\prime }}O_{e}=\frac{O_{e^{\prime }}C}{2}+\frac{C_{1}C_{2}^{2}}{8\times O_{e^{\prime }}C}$$

Gaze direction is calculated in real time by transforming the 2D coordinates of the iris to 3D. This allows us to estimate the direction of gaze by calculating the coordinates of the center of the eye and its relationship to the center of the iris (Figure 5d,e). Two types of tests were therefore performed to validate the gaze estimation methods:

- Detection error test: A bounding box is defined around the head and we check that the eye landmarks remain inside the box throughout the sequence. Any movement outside the box is considered a detection error.
- Landmark Stability Test: The Euclidean distance between landmark positions (eye corners and iris centers) in consecutive frames is calculated to assess stability.

Table 4 presents a comparative analysis of eye landmark detection and stability between different methods. GazeML stands out for its balance between detection accuracy (90%) and stability (0.52 mm), with minimal variability as indicated by its low standard deviation of 0.08 mm. This makes GazeML an optimal choice for our system, as it provides reliable gaze tracking with minimal error, both in terms of detection and stability. Compared to other methods, GazeML is an exemplary choice for real-time gaze tracking applications.

### 2.4. 3D Head Pose Estimation

When focusing on a distant object, the natural movement of the eyes and head requires adjustments in head position. Typically, the head is turned toward the target to reduce eye strain, and often tilted or rotated to improve alignment. Therefore, the relationship between gaze direction and optimal head position makes head pose estimation critical to accurately determine gaze direction [27]. Head position is estimated by capturing and analyzing head motion. The Azure Kinect captures the 3D coordinates of joints in the human body, referred to as the “joint skeleton”, along with the reliability of each detected position. The score assigned to each joint helps with the evaluation of the quality of its detection:

- A score of 0 indicates that the joint is out of range (i.e., too far away from the depth camera).
- A score of 1 indicates that the joint is not observed, probably due to an occlusion, and is therefore predicted.
- A score of 2 represents a moderate level of confidence.
- A score of 3 indicates high confidence in joint detection.

Our method considers the following items:

- A joint is considered well detected if its score is greater than or equal to 2.
- If a hinge point is not well detected, its position is estimated using the previously better detected points. This is possible because the selected head points are considered rigid, i.e., their relative positions remain fixed when subjected to translations and rotations. As a result, any transformation applied to the entire set of points preserves the original geometric properties and relationships between them.

**Figure 6:**
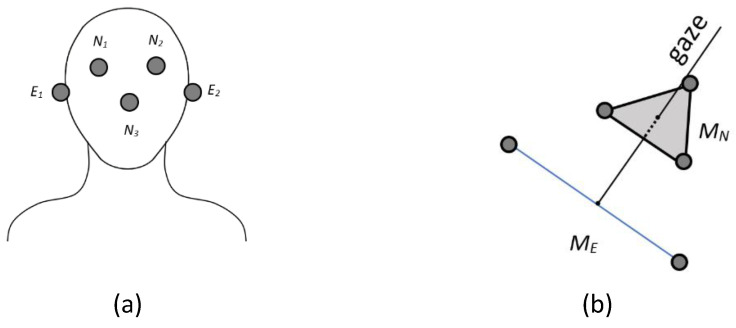
Head position model. (**a**) The points used to estimate the head position: eye right, eye left, ear right, ear left, and nose. (**b**) The 3D coordinates of these points are provided by Azure Kinect.

The following points are used to calculate the head position: eye right $N_{1}$, eye left $N_{2}$, ear right $E_{1}$, ear left $E_{1}$, and nose $N_{3}$ (see Figure 6a). Their 3D representation is shown in Figure 6b. $M_{E}$ represents the midpoint between $E_{1}$ and $E_{2}$ (the center between the ears), while $M_{N}$ is the center of $N_{1}$, $N_{2}$, and $N_{3}$ (the center between the eyes and the nose). This reference was chosen because it contains a point that is lower and more central along the vertical axis of the face. This correction compensates for the upward tilt caused by the difference in height between the eyes and ears, providing a more accurate estimate of head orientation and a more natural reference for the center of the head.

### 2.5. Metrics for Evaluation

The angular error metric is used to evaluate the accuracy of gaze estimation. This metric compares the angular deviation between the estimated and actual gaze vectors. The angular error is given by the following equation:

(5) $$\theta =\cos^{-1}\left(\frac{v_{1}\cdot v_{2}}{|v_{1}\left|\right|v_{2}|}\right)$$

where $v_{1}$ is the estimated gaze direction and $v_{2}$ is the actual gaze direction. Detected child–practitioner interaction periods refer to the time intervals during which the child engages or interacts with the practitioner based on predefined criteria, such as gaze tracking. These periods are identified using manual annotations and their accuracy is assessed using metrics such as IoU and OBOA to ensure that they closely match the actual interaction times [28,29]. The IoU metric is used to measure the accuracy of the overlap between the detected periods and the actual periods (ground truth). Off-By-One Accuracy (OBOA) assesses whether the boundaries (start or end) of the detected interaction periods are sufficiently close to the actual boundaries. In our case, OBOA and IoU can be defined as follows:

(6) $$\mathrm{OBOA}=\frac{1}{D}\sum_{i=1}^{D}\left[|\mathrm{True}\;\mathrm{Duration}-\mathrm{Detected}\;\mathrm{Duration}|\leq 1\right]$$

where *D* is the number of periods in the validation set, and True Duration and Detected Duration refer to the durations of the ground truth and the detected periods, respectively. **IoU** is defined as follows:

(7) $$\mathrm{IoU}=\frac{\min\left(E_{t},E_{p}\right)-\max\left(S_{t},S_{p}\right)}{\max\left(E_{t},E_{p}\right)-\min\left(S_{t},S_{p}\right)}$$

where $S_{t}$ and $E_{t}$ represent the start and end times of the true period (ground truth), and $S_{p}$ and $E_{p}$ represent the start and end times of the detected period. Where $S_{t}$ is the start time of the true period, $E_{t}$ is the end time of the true period, $S_{p}$ is the start time of the detected period, and $E_{p}$ is the end time of the detected period.

## 3. Experimental Results and Discussion

**Figure 7:**
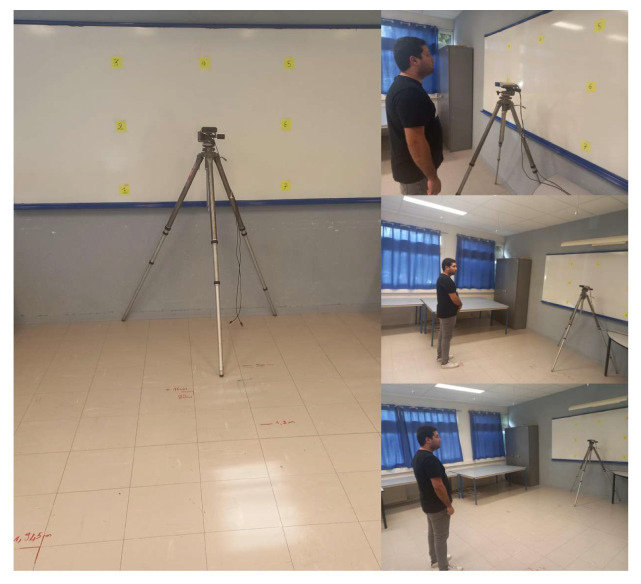
Experimental setup for 3D gaze estimation (target board and Azure Kinect configuration).

This section presents the results of the proposed approach. In order to compare the 3D gaze estimation based on the eye center method and the 3D head pose estimation method, we eliminated the distance condition in our tests. The 3D positions of the eye centers detected using GazeML and the Azure Kinect, as well as the intersection coordinates of the estimated gaze with the target board, were recorded for each frame. To illustrate the experimental setup, Figure 7 shows the scene configuration during the acquisition process. A target board is positioned in front of the participant, with the Azure Kinect camera placed between the participant and the board.

**Figure 8:**
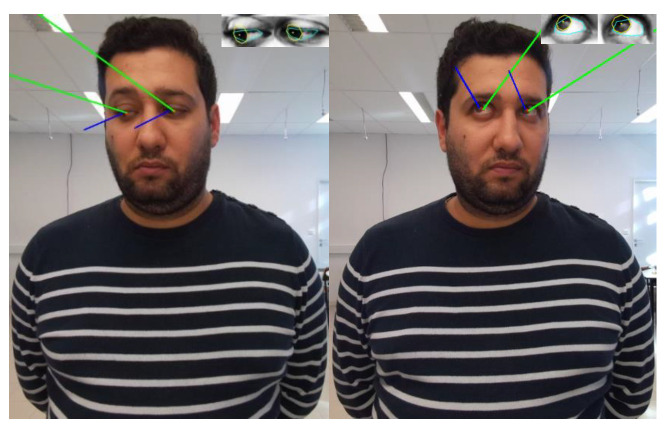
Participant focusing on a target – head orientation (green line) and eye orientation (blue line) for gaze estimation.

Figure 8 shows a participant focusing on a target. In the figure, the green line represents the head orientation, while the blue line follows the eye position, showing the difference between the two estimation approaches.

**Figure 9:**
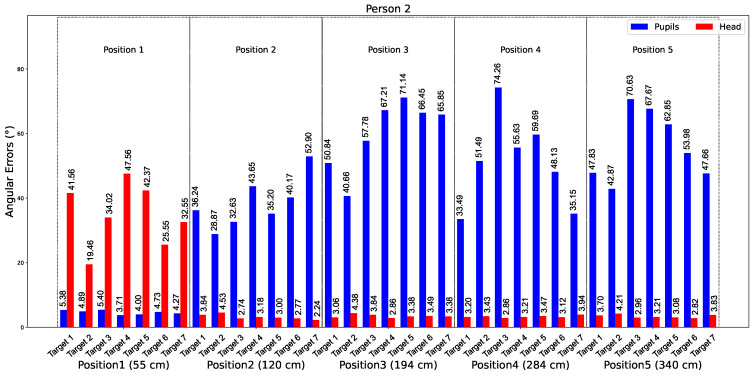
Average angular errors in gaze estimation over ten seconds for Participant 2—comparison of eye-based (blue) and head-based (red) methods across five distances.

Following the protocol described in Section 2.1, the participant focuses on a specific target. The angular error of the gaze direction using the eyes is computed from the 3D coordinates of the eyes, which are captured in 2D by GazeML and converted to 3D as described in Section 2.3. Similarly, for the gaze direction determined with the head, the error is estimated using the 3D coordinates of the eye centers provided by the Azure Kinect system. By combining these errors, our analysis provides a dual-perspective assessment of gaze accuracy, highlighting potential discrepancies between estimates based on eye and head orientation. We evaluated both methods by varying the participants’ positions across five locations to determine the optimal distance for either eye- or head-based gaze estimation. Figure 9 shows the average angular errors over 10 s from tests involving Participant 2. The graph shows data for five distances: 55 cm, 120 cm, 184 cm, 284 cm, and 340 cm. The bars represent the errors for the eyes (in blue) and the head (in red) for all seven targets at each distance.

In Appendix A, we observe that for all participants at distance 1 (55 cm), the estimation of gaze direction using the eyes is more accurate than using the head. However, from position 2 onwards, the head provides more accurate estimates than the eyes. This consistency across participants suggests that variations in physical characteristics, such as eye shape or size, do not affect approach. For Participant 1 at position 1 for target 1, the best detection for the eyes gave an error of 2.57°. For the head, the best detection was achieved by participant 6 at position 2, with an error of 2.06°.

**Figure 10:**
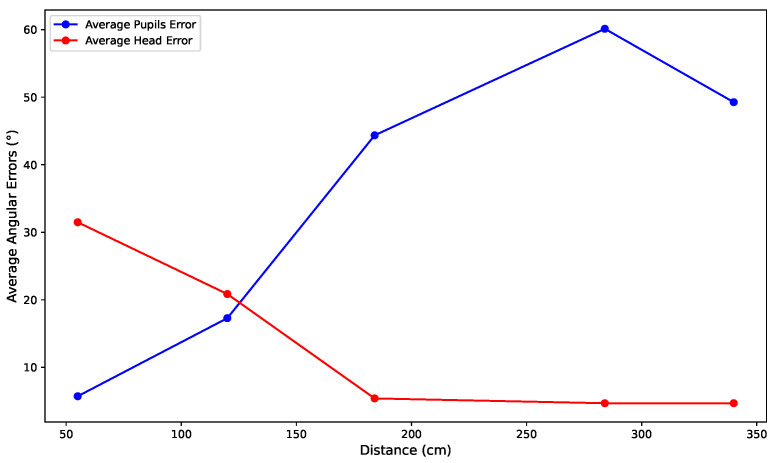
Comparison of gaze estimation accuracy using eye and head landmarks across various distances (55 cm to 340 cm).

Figure 10 shows a graph comparing the average angular errors of gaze estimation using eye and head landmarks at distances ranging from 55 cm to 340 cm. The errors are calculated from gaze data collected for all eight participants, with the average error calculated for each distance.

- The blue curve represents errors using eye landmarks.
- The red curve represents errors using head landmarks.

These results provide insight into the performance of gaze estimation methods. At close distances (e.g., 55 cm), the eye-based approach shows superior accuracy due to the precise detection of eye landmarks. At longer distances (from 120 cm), the head-based method performs better, offering greater reliability and stability. This analysis highlights the complementary nature of the two approaches and their applicability based on specific distance requirements. Table 5 summarizes the angular errors for both methods across different positions and participants. The results confirm that eye-based estimation is superior at close distances, while head-based estimation is better at longer distances. The best results for eye-based estimation were obtained by Subject 3 at position 1 (55 cm), with an angular error of 4.46°, while the best results for head-based estimation were obtained by Subject 2 at position 3 (194 cm), with an angular error of 3.19°.

**Figure 11:**
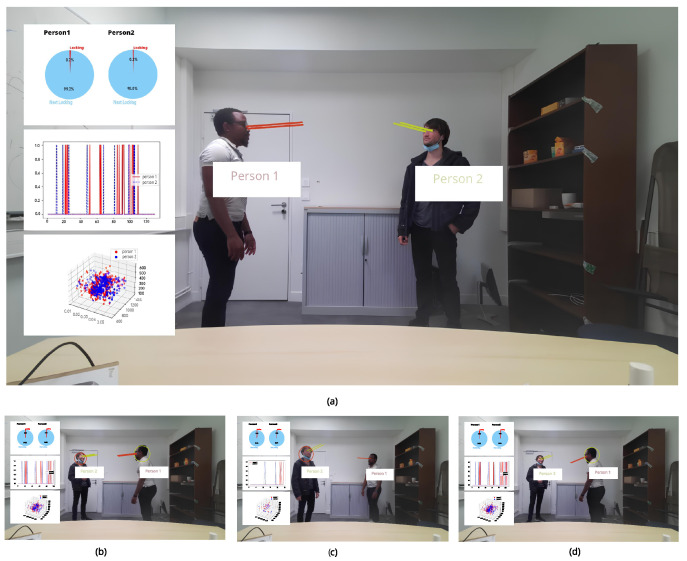
Real-time gaze tracking and mutual gaze detection: (**a**) Overview of the application; (**b**) mutual gaze detection; (**c**) Person 1 gazing at Person 2; (**d**) Person 2 gazing at Person 1.

We tested the method in real time. Figure 11a summarizes the application. On the left, we display the percentage of time each person gazes at the other, shown at the top. In the middle, the peaks represent one person’s gaze toward the other. At the bottom, the trajectory of each person in 3D. We observe the detection of mutual gaze—Figure 11b. Figure 11c shows Person 1 gazing at Person 2, and Figure 11d shows the opposite.

We further tested our approach on recordings to determine when the child was looking at the clinician’s face, comparing this with ground truth annotations. The challenge of skeleton identification, due to Kinect’s limitations, was addressed by training a YOLOv5 [30] model to differentiate between the clinician and the child. This ensured correct identity tracking throughout Sessions 1 and 2. Table 6 shows the results of gaze detection for both interaction sessions (Session 1 and Session 2) during the experiment. It shows the periods during which the children’s gaze intersected with the sphere representing the therapist’s head. Each period is associated with specific moments in time for each session, showing the instances when the children’s gaze was directed toward the therapist. The periods are measured in minutes and seconds. This measure is essential for analyzing the visual attention directed towards the practitioner, which is a key element in evaluating the interactions and serves as a basis for future analyses.

**Figure 12:**
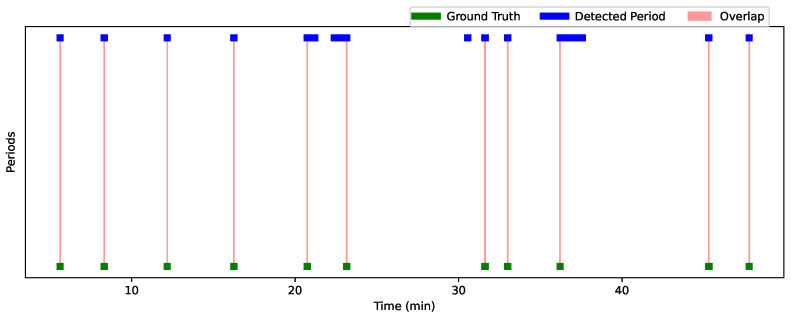
Analysis of gaze detection performance for Session 1—comparison of ground truth (green), detected periods (blue), and overlaps (red).

**Figure 13:**
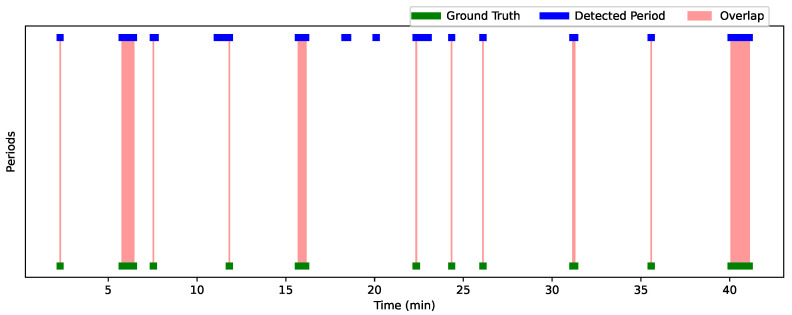
Analysis of gaze detection performance for Session 2—comparison of ground truth (green), detected periods (blue), and overlaps (red).

Figure 12 and Figure 13 show the analysis results for Session 1 and Session 2, allowing a comparison of the performance of the detection approach over these two sessions. The graphs are visualized using a time line, where each bar (or line) represents one period. The width of each bar is proportional to the duration of the detected or actual period. The greater the overlap, the better the performance of the approach. Green periods represent the ground truth moments, while blue periods represent the periods detected by the algorithm. Red areas indicate the overlaps. Periods where the predictions are accurate compared to the ground truth are characterized by large overlaps (extended red areas), while periods with little overlap or detection errors (such as missed or incorrect detections) appear with less overlap or no overlap at all.

**Figure 14:**
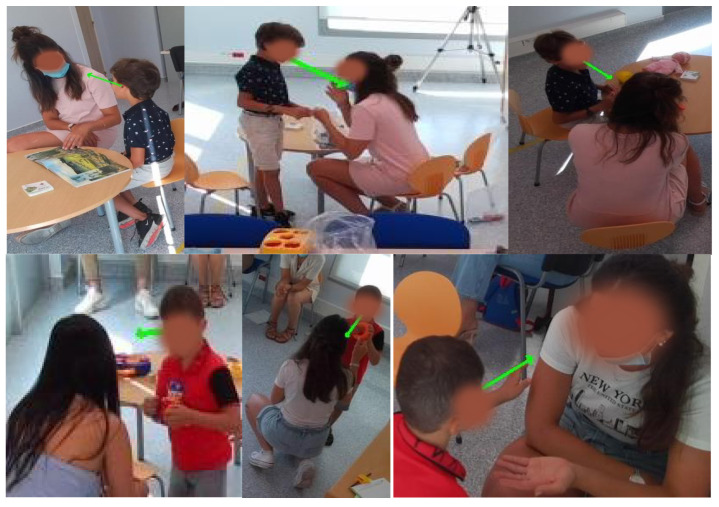
Examples of accurately detected gaze moments for Child 1 and Child 2.

**Figure 15:**
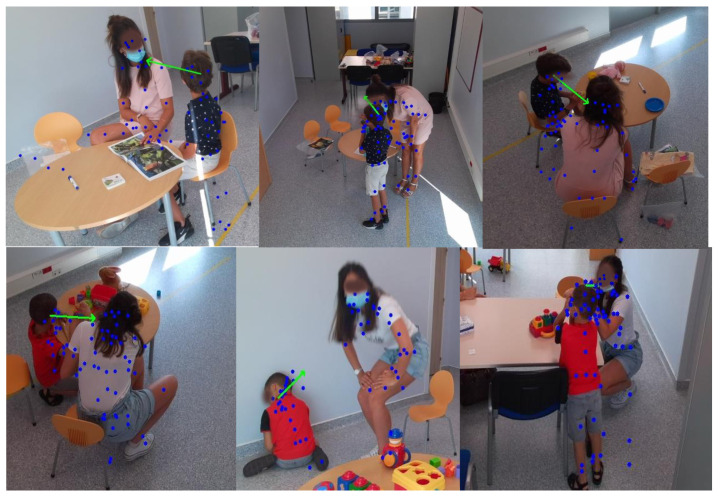
Instances of skeleton overlap and sideways facing during close profile interactions.

Table 7 summarizes the interaction results for Child 1 and Child 2, showing the overlap periods along with their corresponding IoU and OBOA values. For Child 1, the average IoU is 0.67 and the average OBOA is 0.73. Child 2 has a higher average IoU of 0.75, with the same average OBOA of 0.73. This indicates a relatively high degree of overlap between the detected periods and the truth, with some instances of low overlap (such as 20:44–20:45, with an IoU of 0.0357). Figure 14 shows sequences where we detected good results for Child 1 and Child 2. A closer analysis of the incorrectly detected periods shows that these errors occurred mainly when the child was facing sideways or in profile relative to the four cameras. In addition, we observed that when the profiles of the child and the practitioner were close, their skeletons overlapped (see Figure 15). In these situations, the child’s face was not detected in any of the cameras, and facial landmarks were poorly estimated, even when the estimation scores were relatively good. This resulted in the system detecting periods when there was no gaze intersection.

Despite these detection errors, the high IoU and OBOA scores (e.g., 1.0 for periods such as 5:37–5:38 for Child 1 and 47:46–47:47 for Child 2) suggest that the detection approach is generally effective in reliably identifying gaze detection moments. In summary, although some periods show temporal variations, all detections are present, indicating that the system is tracking gaze detection moments, with performance close to the truth in most cases. Our method successfully detected all periods when the child was looking at the participant. Despite these false detections, the strength of our method lies in its ability to accurately identify the moments when the child is looking at the participant, which is of great value to practitioners. By automatically detecting these moments, we save a considerable amount of time compared to manually searching through the video to identify when the child is focused on the practitioner. This makes our method very useful for tasks that require efficient gaze estimation.

As shown in Table 8, our method has significant advantages over other existing datasets and methods. By using precise RGB-D measurements over a range of 55–340 cm, we provide a more flexible and accurate approach to head and eye tracking. This extended range allows for greater versatility in various real-world applications, surpassing many existing datasets that are limited by smaller distance ranges. In addition, our method’s ability to handle both head and eye annotations across all orientations further enhances its robustness and adaptability compared to more constrained methods. This makes our approach a more comprehensive solution for a wider range of tracking scenarios, offering improved performance and accuracy across different distances and orientations.

In accordance with this, we adapt the Eyediap and MPIIGaze databases to better meet the requirements of our method. For MPIIGaze, we implement additional functionalities, in particular the detection of the iris in 2D and the computation of its position in 3D, since this information is not available in the original dataset. In addition, we extract nose landmarks in 3D to improve the accuracy of head and gaze estimation. For Eyediap, a similar adaptation process is underway to ensure compatibility with our framework. In addition, we are preparing five new datasets specifically adapted for children with autism. These datasets aim to capture unique gaze behaviors as well as person and object tracking dynamics. As part of this effort, we are correcting errors in person identification and refining skeletal joint positions, particularly with respect to inaccuracies observed in skeletal recognition. This preparation allows us to robustly evaluate and optimize our approach in real-world scenarios, particularly for applications involving children with ASD.

## 4. Conclusions

This study presents a new eye-tracking method using Azure Kinect to measure gaze direction and head orientation in children with autism spectrum disorder (ASD). Using 3D anatomical landmarks, the method significantly improves the accuracy of gaze estimation, especially in real-world environments where traditional approaches are challenging. Experimental results show angular errors as low as 2.06° in some scenarios, confirming the robustness of the method at different distances and head positions. The integration of eye tracking and head tracking proves to be complementary, with the former excelling at short distances and the latter offering greater reliability at long distances. By enabling gaze tracking during social interactions, such as a child’s attention to a clinician, this approach improves clinical assessments and provides valuable information about social intentions. It holds promise for improving diagnostic and therapeutic interventions where gaze behavior is a key element of social communication. For the future, we are working to adapt existing databases and prepare new datasets to optimize the proposed method for children with autism. These initiatives aim to improve the robustness and applicability of our approach in real-world contexts, particularly for ASD applications.

## Figures and Tables

**Table 1:** Three-dimensional coordinates of the seven targets captured by Kinect.

Coordinates	Target 1	Target 2	Target 3	Target 4	Target 5	Target 6	Target 7
X coordinates (cm)	603.5	603.5	603.5	0	−576.5	−576.5	−576.5
Y coordinates (cm)	427.3	0	−432.7	−432.7	−432.7	0	432.7
Z coordinates (cm)	−398.3	−398.3	−398.3	−398.3	−398.3	−398.3	−398.3

**Table 2:** Participant positions and corresponding X, Y, and Z coordinates.

Coordinates	Position 1	Position 2	Position 3	Position 4	Position 5
X Coordinates (cm)	0	0	76	−75	0
Y Coordinates (cm)	143	143	143	143	143
Z Coordinates (cm)	55	120	194	284	340

**Table 3:** Gaze durations and interaction periods for child–clinician engagement.

Periods	Session 1 (Start–End)	Session 2 (Start–End)
**Total Video**	**53 min 55 s**	**43 min 31 s**
Period 1	5 min 37 s–5 min 38 s	2 min 17 s–2 min 18 s
Period 2	8 min 19 s–8 min 20 s	5 min 47 s–6 min 26 s
Period 3	12 min 10 s–12 min 11 s	7 min 32 s–7 min 33 s
Period 4	16 min 15 s–16 min 16 s	11 min 49 s–11 min 50 s
Period 5	20 min 44 s–20 min 45 s	15 min 42 s–16 min 8 s
Period 6	23 min 9 s–23 min 10 s	22 min 20 s–22 min 22 s
Period 7	31 min 36 s–31 min 38 s	24 min 20 s–24 min 21 s
Period 8	33 min 0 s–33 min 1 s	26 min 6 s–26 min 7 s
Period 9	36 min 12 s–36 min 13 s	31 min 10 s–31 min 17 s
Period 10	45 min 18 s–45 min 20 s	35 min 34 s–35 min 35 s
Period 11	47 min 46 s–47 min 47 s	40 min 4 s–40 min 11 s

**Table 4:** Comparative analysis of eye landmark detection and stability across different methods.

Method	Detection (%)	Stability (mm)	Stability Std. Dev. (mm)
OpenFace [24]	89%	0.91	0.15
GazeML	90%	0.52	0.08
MediaPipe Face Mesh [25]	93%	0.84	0.12
Face-Alignment [26]	91%	0.99	0.18

**Table 5:** Summary of angular errors for eye-based and head-based gaze estimation across positions and participants.

Participants	Position 1 (55 cm)	Position 2 (120 cm)	Position 3 (194 cm)	Position 4 (284 cm)	Position 5 (340 cm)
Participant 1	Pu.: 6.67 He.: 26.79	Pu.: 34.50 He.: 10.84	Pu.: 65.09 He.: 3.74	Pu.: 43.09 He.: 4.92	Pu.: 51.98 He.: 3.78
Participant 2	Pu.: 4.63 He.: 34.72	Pu.: 38.52 He.: 3.19	Pu.: 59.99 He.: 3.48	Pu.: 51.12 He.: 3.32	Pu.: 56.21 He.: 3.40
Participant 3	Pu.: 4.46 He.: 33.44	Pu.: 39.20 He.: 4.74	Pu.: 58.50 He.: 4.04	Pu.: 46.02 He.: 4.89	Pu.: 33.43 He.: 5.55
Participant 4	Pu.: 5.30 He.: 40.06	Pu.: 47.50 He.: 5.25	Pu.: 65.33 He.: 5.11	Pu.: 45.28 He.: 4.13	Pu.: 58.11 He.: 4.62
Participant 5	Pu.: 4.54 He.: 24.40	Pu.: 46.73 He.: 4.40	Pu.: 66.03 He.: 3.71	Pu.: 58.03 He.: 3.92	Pu.: 53.01 He.: 7.94
Participant 6	Pu.: 6.22 He.: 30.22	Pu.: 58.78 He.: 3.96	Pu.: 54.33 He.: 4.03	Pu.: 53.70 He.: 4.54	Pu.: 58.39 He.: 4.41
Participant 7	Pu.: 6.61 He.: 36.51	Pu.: 44.82 He.: 5.87	Pu.: 57.32 He.: 9.07	Pu.: 52.21 He.: 5.19	Pu.: 54.82 He.: 4.19
Participant 8	Pu.: 7.30 He.: 25.70	Pu.: 44.77 He.: 4.90	Pu.: 54.39 He.: 4.28	Pu.: 44.63 He.: 6.62	Pu.: 50.66 He.: 4.23

**Table 6:** Gaze detection results for interaction sessions (Session 1 and Session 2)—periods of gaze intersection child–clinician.

Periods	Session 1 Start–End	Session 2 Start–End
**Total Video**	**53 min 55 s**	**43 min 31 s**
Period 1	5 min 37 s–5 min 38 s	2 min 17 s–2 min 18 s
Period 2	8 min 19 s–8 min 20 s	5 min 47 s–6 min 26 s
Period 3	12 min 10 s–12 min 11 s	7 min 32 s–7 min 39 s
Period 4	16 min 15 s–16 min 16 s	11 min 8 s–11 min 50 s
Period 5	20 min 44 s–21 min 12 s	15 min 42 s–16 min 8 s
Period 6	22 min 23 s–23 min 10 s	18 min 20 s–18 min 29 s
Period 7	30 min 33 s–30 min 34 s	20 min 5 s–20 min 6 s
Period 8	31 min 36 s–31 min 38 s	22 min 20 s–23 min 2 s
Period 9	33 min 0 s–33 min 1 s	24 min 20 s–24 min 21 s
Period 10	36 min 12 s–37 min 34 s	26 min 6 s–26 min 7 s
Period 11	45 min 17 s–45 min 19 s	31 min 10 s–31 min 17 s
Period 12	47 min 46 s–47 min 47 s	35 min 34 s–35 min 35 s
Period 13	/	40 min 4 s–41 min 6 s

**Table 7:** Summary of interaction results for Child 1 and Child 2—overlap periods with IoU and OBOA values.

Overlap Child 1 Start–End	IoU (Child 1)	OBOA (Child 1)	Overlap Child 2 Start–End	IoU (Child 2)	OBOA (Child 2)
5:37–5:38	1.000000	1	2:17–2:18	1.000000	1
8:19–8:20	1.000000	1	5:47–6:26	1.000000	1
12:10–12:11	1.000000	1	7:32–7:33	0.142857	0
16:15–16:16	1.000000	1	11:49–11:50	0.023810	0
20:44–20:45	0.035714	0	15:42–16:08	1.000000	1
23:09–23:10	0.021277	0	22:20–22:22	0.047619	0
31:36–31:38	1.000000	1	24:20–24:21	1.000000	1
33:00–33:01	1.000000	1	26:06–26:07	1.000000	1
36:12–36:13	0.012195	0	31:10–31:17	1.000000	1
45:18–45:19	0.333333	1	35:34–35:35	1.000000	1
47:46–47:47	1.000000	1	40:04–41:06	1.000000	1

**Table 8:** Comparison of our method with existing datasets and methods.

Dataset or Methods	RGB or RGB-D	Distance Limits	Annotations	Orientations
BIWI [31]	RGB-D	100 cm	Head	All
ICT 3D Head pose [32]	RGB-D	100 cm	Head	All
Deep Head Pose [33]	RGB-D	200–800 cm	Head	All
EyeDiap	RGB-D	80–120 cm	Head-Eyes	Frontal
RT-GENE [34]	RGB-D	80–280 cm	Head-Eyes	All
MPII Gaze	RGB	40–60 cm	Head-Eyes	Frontal
ETH-XGaze [35]	RGB	100 cm	Head-Eyes	Frontal
Our Method	Precise RGB-D metering	55–340 cm	Head-Eyes	All

**Figure A1:**
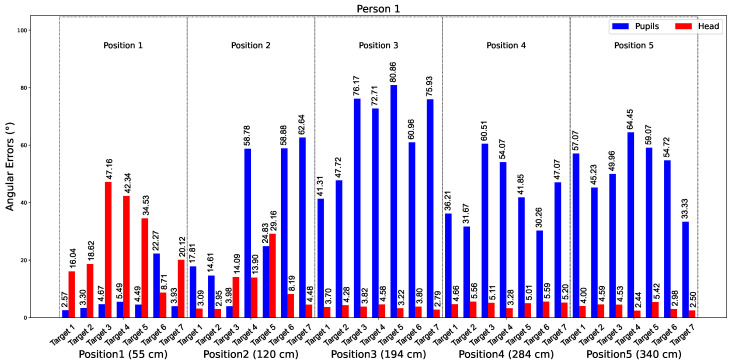

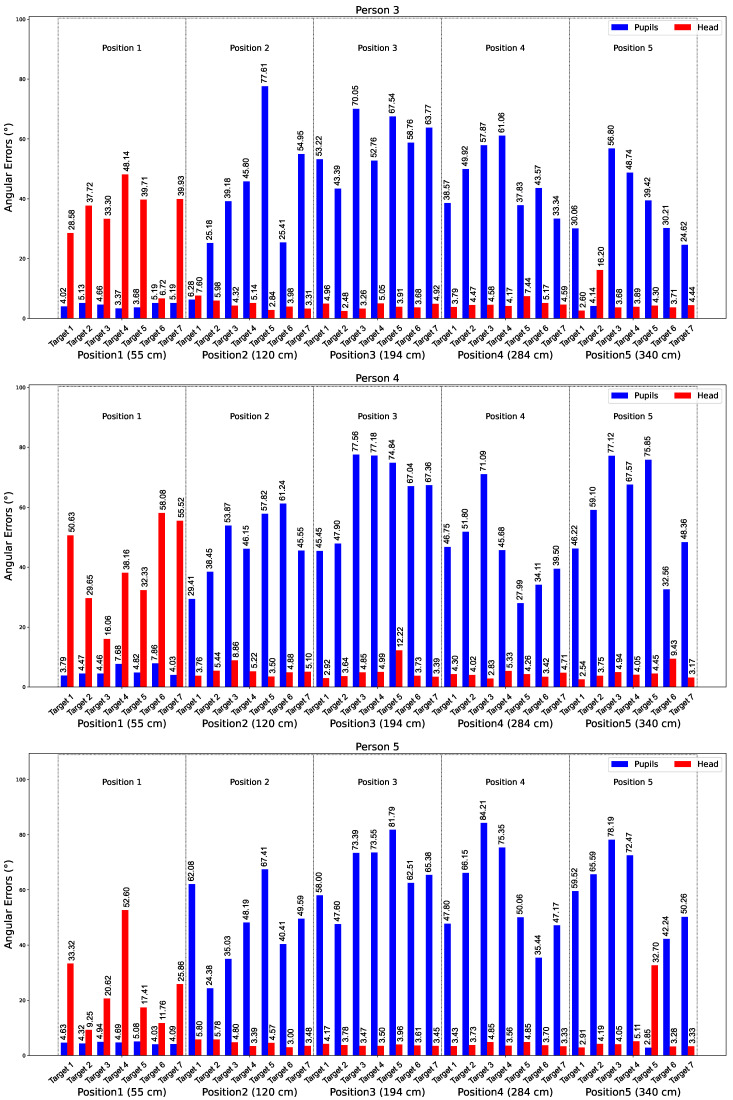

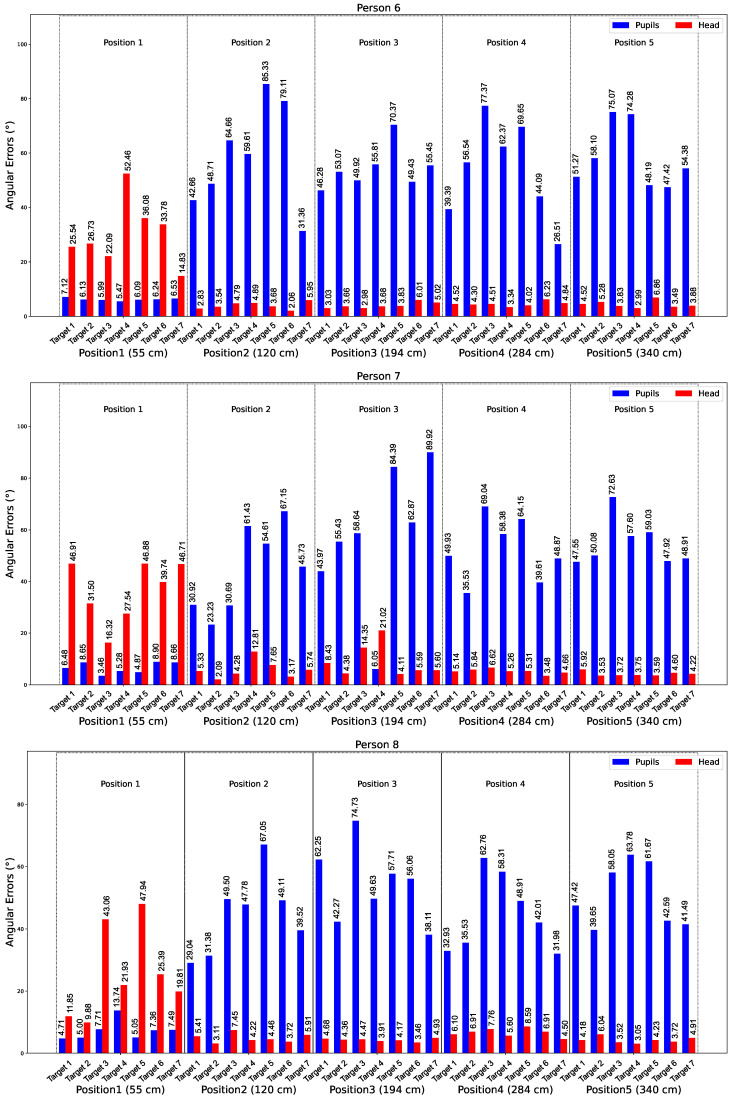
Comparison of gaze estimation accuracy using eyes and head across participants and distances.

## Data Availability

ACTIVIS dataset: This dataset is subject to ethical restrictions and cannot be shared publicly. For access requests, please contact the corresponding author. The EYELIS dataset with gaze direction and head orientation will be published soon and will be made available in a public repository. The link will be provided once the publication is available.

## Abbreviations

The following abbreviations are used in this manuscript:

RGB	Red, Green, Blue
RGB-D	Red, Green, Blue + Depth
VR	Virtual Reality
HCI	Human–Computer Interaction
ASD	Autism Spectrum Disorder
ADOS	Autism Diagnostic Observation Schedule
PECS	Picture Exchange Communication Systems
EOG	Electro-oculography
TOF	Time-of-flight
IoU	Intersection over Union
OBOA	Off-By-One Accuracy
